# Prevalence and Sociodemographic Determinants of Hypertension History among Women in Reproductive Age in Ghana

**DOI:** 10.1155/2016/3292938

**Published:** 2016-04-20

**Authors:** Samuel H. Nyarko

**Affiliations:** Department of Population and Behavioural Sciences, School of Public Health, University of Health and Allied Sciences, Hohoe, Ghana

## Abstract

*Background*. Hypertension is a global health problem. Yet, studies on hypertension rarely focus on women in Ghana. The purpose of this study is to ascertain the prevalence and sociodemographic determinants of hypertension history among Ghanaian women in reproductive age. *Methods*. This study used data from the 2014 Ghana Demographic and Health Survey. Bivariate and logistic regression analyses were carried out to ascertain the prevalence and determinants of hypertension history among the respondents. *Results*. The study found that the overall prevalence of hypertension history among the respondents was 7.5%; however, there were vast variations within most of the sociodemographic categories. Age, level of education, marital status, work status, and wealth status had a significant relationship with hypertension history among the respondents. Women in advanced age groups, highly educated, married, and widowed/divorced/separated, nonworking women, and women from wealthy households were at higher risk of having hypertension history. *Conclusion*. Myriads of sociodemographic factors determine the hypertension history of women in Ghana. It is therefore essential to target medical and psychosocial hypertension interventions at Ghanaian women in the higher risk groups.

## 1. Background

Hypertension is the constant pumping of blood through blood vessels with excessive force [1]. It is a major public health problem that is associated with relatively low levels of awareness, drug treatment, and blood pressure control [2, 3]. It is a “silent killer” because it often has no warning signs or symptoms, and many people do not realize they have it [1]. It can cause serious damage to health by hardening the arteries, decreasing the flow of blood and oxygen to the heart which in turn can cause chest pain, heart failure, heart attack, and stroke [1].

Worldwide, the overall prevalence of hypertension in adults aged 25 and above in 2008 was around 40 percent. Even though the proportion of the world's population with hypertension decreased slightly between 1980 and 2008, because of population growth and ageing, the number of people with uncontrolled hypertension rose from 600 million to nearly 1 billion between 1980 and 2008 [4]. Hypertension is estimated to cause 7.5 million deaths, about 12.8 percent of the total of all global deaths, accounting for 57 million disability adjusted life years (DALYS) [4]. Across the World Health Organisation regions, the prevalence of hypertension was highest in Africa, where it was 46 percent for both sexes. The lowest prevalence was in the Americas at 35 percent for both sexes [4].

In Ghana, the prevalence of adult hypertension has been found to be consistently high in both urban and rural areas ranging from 19 to 48 percent [5]. The awareness, treatment experience, and effective control of hypertension were also found to be low [3, 5], with treatment levels ranging from 6.9 to 52.5 percent while control levels range from 1.7 to 12.7 percent [5]. Hypertension has been found to be a significant cause of renal and heart failure in Ghana [6, 7] and has been one of the root causes of higher levels of cardiovascular diseases in Africa [8]. It is quite obvious that inadequate population-based studies on hypertension have been conducted in Ghana, with virtually all of them focused on subpopulations. It has also been observed that albeit population-based prevention strategies including reduction in salt intake and integration of hypertension care into primary care may prove beneficial, the determinants of hypertension remain to be ascertained [2]. This paper therefore sought to ascertain the prevalence and sociodemographic determinants of hypertension history among women in reproductive age (15–49) in Ghana.

## 2. Materials and Methods

Data for this study were obtained from the 2014 Ghana Demographic and Health Survey (2014 GDHS), implemented by the Ghana Statistical Service (GSS), the Ghana Health Service (GHS), and the National Public Health Reference Laboratory (NPHRL) of the GHS. It is the sixth and latest in a series of population and health surveys conducted in Ghana as part of the global Demographic and Health Surveys (DHS) Program. A nationally representative survey of 9,396 women aged 15–49 from 11,835 households was conducted [9]. Ethical approval for the survey was catered for by the Ghana Health Service Ethical Review Committee; therefore, further ethical approval is not applicable in this case.

The outcome variable of the study is history of hypertension among women. In determining this, respondents were asked about their hypertension history, as to whether they had ever been told by a doctor or any health personnel that they had high blood pressure. This generated a binary response from the respondents in terms of yes or no. The independent variables therefore were a number of sociodemographic factors that may have the potential to influence hypertension history of respondents. These comprised age, education level, religious affiliation, ethnicity, wealth, marital and work status, and type and region of residence.

The data were extracted and processed using Stata version 13. Some variables were therefore recoded to suit the purpose of the study. To adjust for clustering during sampling and for that reason ensure national representation of the findings, all results were weighted using the generated sample weight for the dataset. In this sense, prevalence of hypertension history was computed and presented in the form of proportions by selected sociodemographic characteristics of respondents. In order to ascertain the sociodemographic factors that determined hypertension history among the respondents, logistic regression analysis was applied. Consequently, odds ratios, *P* values, and confidence intervals were computed and presented in Tables 1 and 2 for analysis and discussion.

## 3. Results

### 3.1. Sociodemographic Characteristics of Respondents

Table 1 presents a summary of the sociodemographic characteristics of respondents and prevalence of hypertension history. As indicated in the table, more than one-third (34.2%) of the respondents who participated in the study were from 20 to 29 years while about one-fifth (20.1%) were from 40 to 49 years and 17.3 percent were from 15 to 19 years. About 63 percent had secondary education or higher while 17.8 percent had primary education.

With regard to religious affiliation, about 80 percent were Christians and 15.2 percent were Muslims while only 2 percent were traditionalists. More than half (52.4%) were Akans while 14.8 percent were Mole-Dagbanis and less than one-tenth (7.7%) were Ga-Dangmes. In terms of household wealth, about 46 percent were from rich households while about one-third (33.5%) were from poor households. Also, about 57 percent were married or living together while 33 percent were never married or living together. The majority (73.4%) were working while 26.6 percent were not working. For type of residence, more than half (53.7%) were from the urban settings while 46.3 were from rural settings. In relation to region of residence, about one-fifth (20.2%) and close to one-fifth (19.1%) were from the Greater Accra and Ashanti Regions, respectively, while only 3.8 and 2.3 percent were from the Upper East and West regions, respectively.

### 3.2. Prevalence of Hypertension History among Women in Reproductive Age

During the survey, respondents were asked whether they had been told by a doctor or any health professional that they had high blood pressure. A summary of this has been presented by the sociodemographic characteristics of respondents (Table 1). Overall, 7.5 percent of the respondents reported ever being told by a health professional that they had hypertension. The highest percentage of hypertension history was reported among women aged 40–49 (18.4%) while the lowest percentage was reported among women aged 15–19.

Women who had primary school education reported higher percentage (7.8%) of hypertension history than their counterparts who had secondary school education or higher (7.7%) and no formal education (6.6%). The prevalence of hypertension history was also highest among Christian women (7.6%) but lowest among traditional women (5.4%). With regard to ethnicity, the highest percentages were reported among Ga-Dangme (12.6%) and Ewe women (10.6%) while the lowest were reported among Mole-Dagbani (6.2%) and other groups of women (4.3%). Further, the percentage of hypertension history was highest among women from rich households but lowest among their counterparts from poor households. Apparently, the prevalence of hypertension increased greatly with wealth status of the respondents. Working women at the time of the survey also reported higher percentage (8.5%) of hypertension history than their counterparts who were not working (4.7%). There were also some spatial variations in hypertension history among the respondents. For instance, women from urban settings reported higher percentage (9.7%) of hypertension history than those from rural settings (5.0%). Also, women from the Greater Accra Region (13.1%) reported the highest prevalence of hypertension history, followed by women from the Volta Region (9.3%) and Eastern Region (8.3%). However, the lowest prevalence was reported among women from the Upper West Region (3.2%).

### 3.3. Determinants of Hypertension History among Women in Reproductive Age

In order to ascertain the potential determinants of hypertension history among the respondents, a logistic regression analysis including odds ratios was applied (Table 2). A cursory look at the table shows that age of woman, level of education, and marital, work, and wealth status had a significant relationship with hypertension history, unlike religious affiliation, ethnicity, and place and region of residence. With regard to risk of hypertension history, it was found that the odds were higher among women aged 40–49 (OR = 34.41), 30–39 (OR = 13.11), and 20–29 (OR = 5.14) compared to women aged 15–19. The odds were also higher among women who had primary school education (OR = 1.45) and secondary school or higher education (OR = 1.48) compared to women who had no formal education.

Women who were married or living together (OR = 2.08) and women who were widowed, divorced, or separated (OR = 2.21) also had higher odds of hypertension history compared to their counterparts who were never married or living together. The odds were however lower among working women (OR = 0.77) compared to nonworking women. Concerning wealth status, women from rich (OR = 2.41) and average (OR = 1.69) households had higher odds of hypertension history compared to their counterparts from poor households. Even though region of residence, on the whole, had no significant relationship with hypertension history, the Central (*P* = 0.010), Greater Accra (*P* = 0.007), and Volta (*P* = 0.043) Regions had significant influence on the regional variations among the respondents.

## 4. Discussion

In this study, about 8 percent of women in reproductive age in Ghana were found to have hypertension history. Age, education, and marital, work, and wealth status were found to be associated with hypertension history among the respondents. In view of this, the risk of hypertension history had increased considerably with increasing age among respondents. A number of studies across the globe have also found that increasing age is a determining factor of hypertension status among respondents [5, 10–14]. The reason for the high risk of hypertension among women in advanced age may be subject to a number of interpretations. For instance, unlike younger women, women in advanced age groups may not have the physical energy, the cardiovascular resistance, or the zeal needed to perform regular aerobic exercises which may help maintain optimum blood pressure level. Besides, some women in advanced age groups may prefer sedentary lifestyles or jobs which in itself may be a risk factor for hypertension [13], while some may even be prone to thinking too much about “trivial circumstances” in their lives. These among others may likely render women in advanced age groups more susceptible to high blood pressure or hypertension.

Level of education was also found to have a significant influence on hypertension history among the respondents. Thus, the risk of hypertension history increased marginally with increasing level of education among the respondents. It has also been reported by a number of studies that educational level or literacy is significantly associated with hypertension [13–16]. Like this study, some of these studies have found higher risk of hypertension among the highly educated compared to the uneducated while others have also reported the reverse. However, none of them has been able to provide possible reasons for their findings. This may be as a result of the fact that the factors that made highly educated women more susceptible to hypertension may be unclear and for that matter difficult to assert.

The study further found that married or cohabiting women and widows, divorcees, or separated women were more likely to have hypertension history than women who were never married. It was observed that existing studies that examined determinants of hypertension among a number of different populations hardly considered marital status of respondents. Even though it is quite unclear why married and widowed, divorced, or separated women were more susceptible to hypertension history, it is possible that this may be due to the inevitable “vicissitudes of marriage.”

Additionally, working women were found to be less likely to have hypertension history compared to their counterparts who were not working. Even though the prevalence of hypertension history was higher among working women, the risk of hypertension history was higher among nonworking women. The influence of working status on the hypertension history of respondents has been scarcely investigated by studies. Nevertheless, it may be fair to presume that higher risk of hypertension history among nonworking women may be due to “inactivity” or “sedentary life” [13, 16, 17] and its concomitant social and economic pressures.

The effect of household wealth status on hypertension history has also been established in this study. The risk of hypertension history was therefore found to increase appreciably with increasing household wealth status of respondents. A number of reports have indicated that household wealth index or socioeconomic status has a significant relationship with hypertension history among people [12, 13, 18]. Like this study, some reports have indicated a positive relationship between wealth index and hypertension whereas others have found a negative relationship between the two. However, it is not clear which factors might have subjected women from rich households to higher risk of hypertension history in this study. Perhaps, one of the reasons may be that women from households with higher wealth index in Ghana may mostly be using vehicles or gadgets for their daily duties instead of performing physical activities such as regular walking and manual work which may be a protective factor against hypertension. This may render women from rich households vulnerable to hypertension compared to their poor counterparts.

## 5. Conclusion

Hypertension history, even though not quite high on the whole, is still a health problem among women in reproductive age in Ghana, following the vast variations found within the subcategories of the sociodemographic characteristics of respondents. Age of respondent, level of education, marital status, work status, and household wealth status were therefore the sociodemographic factors that determined hypertension history among women in reproductive age group in Ghana for the study period. Consequently, it becomes necessary to target medical and psychosocial hypertension interventions towards Ghanaian women who are in advanced age groups, highly educated, married, widowed/divorced/separated, and not working as well as women from wealthy households.

## Figures and Tables

**Table 1:** Prevalence of hypertension history by sociodemographic characteristics.

Sociodemographic characteristics	Percentage with hypertension history	Number and percent of women
Age		
15–19	0.4	1,625 (17.3)
20–29	3.2	3,217 (34.2)
30–39	9.2	2,667 (28.4)
40–49	18.4	1,887 (20.1)
Education level		
No formal education	6.6	1,792 (19.1)
Primary education	7.8	1,672 (17.8)
Secondary/higher education	7.7	5,932 (63.1)
Religious affiliation		
Christian	7.6	7,529 (80.1)
Muslim	7.3	1,424 (15.2)
Traditionalist	5.4	189 (2.0)
No religion	5.7	254 (2.7)
Ethnicity		
Akan	7.0	4,920 (54.4)
Ga-Dangme	12.6	727 (7.7)
Ewe	10.6	1,267 (13.5)
Mole-Dagbani	6.2	1,389 (14.8)
Others	4.3	1,093 (11.6)
Wealth status		
Poor	4.1	3,147 (33.5)
Middle	7.0	1,938 (20.6)
Rich	10.3	4,311 (45.9)
Marital status		
Never married	1.7	3,094 (33.0)
Married/living together	9.7	5,321 (56.6)
Widowed/divorced/separated	13.5	981 (10.4)
Work status		
Not working	4.7	2,495 (73.4)
Working	8.5	6,901 (26.6)
Type of residence		
Urban	9.7	5,051 (53.7)
Rural	5.0	4,345 (46.3)
Region		
Western	5.6	1,038 (11.0)
Central	3.3	937 (10.0)
Greater Accra	13.1	1,898 (20.2)
Volta	9.3	720 (7.7)
Eastern	8.3	877 (9.3)
Ashanti	6.6	1,798 (19.1)
Brong Ahafo	5.5	769 (8.2)
Northern	5.6	786 (8.4)
Upper East	4.1	358 (3.8)
Upper West	3.2	215 (2.3)
Total 15–49	7.5	9,396

**Table 2:** Logistic regression of hypertension history among women aged 15–49.

Variables	Odds ratio (OR)	*P* value	95% conf. interval
Age of woman			
15–19 (Ref.)	1		
20–29	5.14	0.000^*∗∗∗*^	2.19–2.75
30–39	13.11	0.000^*∗∗∗*^	1.42–1.71
40–49	34.41	0.000^*∗∗∗*^	1.14–3.71
Level of education			
No formal education (Ref.)	1		
Primary	1.45	0.023^*∗*^	1.05–2.09
Secondary/higher	1.48	0.026^*∗*^	1.04–2.02
Religious affiliation			
Christian (Ref.)	1		
Muslim	1.31	0.105	0.94–1.84
Traditional/spiritualist	0.83	0.676	0.35–1.95
No religion	0.76	0.390	0.41–1.40
Ethnicity			
Akan (Ref.)	1		
Ga/Dangme	1.16	0.427	0.79–1.69
Ewe	1.32	0.119	0.92–1.88
Mole-Dagbani	0.13	0.596	0.70–1.82
Other	0.77	0.250	0.50–1.19
Marital status			
Never married (Ref.)	1		
Married/living together	2.08	0.001^*∗∗∗*^	1.36–3.18
Widowed/divorced/separated	2.21	0.001^*∗∗∗*^	1.35–3.58
Work status			
Not working (Ref.)	1		
Working	0.77	0.050^*∗*^	0.58–1.02
Wealth status			
Poor	1		
Average	1.69	0.001^*∗∗∗*^	1.22–2.33
Rich	2.41	0.000^*∗∗∗*^	1.66–3.51
Place of residence			
Urban (Ref.)	1		
Rural	0.90	0.481	0.69–1.19
Region of residence			
Western	1		
Central	0.54	0.010^*∗∗*^	0.34–0.86
Greater Accra	1.73	0.007^*∗∗*^	1.16–2.60
Volta	1.65	0.043^*∗*^	1.01–2.68
Eastern	1.40	0.101	0.93–2.09
Ashanti	0.94	0.781	0.63–1.41
Brong Ahafo	1.16	0.499	0.74–1.83
Northern	1.68	0.052	0.99–2.84
Upper East	1.18	0.556	0.67–2.09
Upper West	0.82	0.538	0.44–1.51

Ref. = reference category.

^*∗*^ *P* ≤ 0.05; ^*∗∗*^ *P* ≤ 0.01; and ^*∗∗∗*^ *P* ≤ 0.001.

## References

[1] World Health Organization (2011). *Hypertension*.

[2] Amoah A. G. B. (2003). Hypertension in Ghana: a cross-sectional community prevalence study in Greater Accra. *Ethnicity & Disease*.

[3] Addo J., Agyemang C., Smeeth L. A., de-Graft Aikins A., Edusei A. K., Ogedegbe O. (2012). A review of population-based studies on hypertension in Ghana. *Ghana Medical Journal*.

[4] World Health Organization (2016). *Raised Blood Pressure: Situation and Trends*.

[5] Bosu W. K. (2010). Epidemic of hypertension in Ghana: a systematic review. *BMC Public Health*.

[6] Plange-Rhule J., Phillips R., Acheampong J. W., Saggar-Malik A. K., Cappuccio F. P., Eastwood J. B. (1999). Hypertension and renal failure in Kumasi, Ghana. *Journal of Human Hypertension*.

[7] Owusu I. (2006). Causes of heart failure as seen in Kumasi, Ghana. *The Internet Journal of Third World Medicine*.

[8] Cooper R. S., Amoah A. G. B., Mensah G. A. (2003). High blood pressure: the foundation for epidemic cardiovascular disease in African populations. *Ethnicity and Disease*.

[9] Ghana Statistical Service (GSS) (2015). *Ghana Demographic and Health Survey 2014*.

[10] van den Berg N., Meinke-Franze C., Fiss T., Baumeister S. E., Hoffmann W. (2013). Prevalence and determinants of controlled hypertension in a German population cohort. *BMC Public Health*.

[11] Ugwuja E. I., Ezenkwa U. S., Nwibo A. N., Ogbanshi M., Idoko O., Nnabu R. (2015). Prevalence and determinants of hypertension in an agrarian rural community in Southeast Nigeria. *Annals of Medical and Health Sciences Research*.

[12] Alam M., Soni G. P., Jain K. K., Verma S., Panda P. (2015). Prevalence and determinants of hypertension in elderly population of Raipur city, Chhattisgarh. *International Journal of Research in Medical Sciences*.

[13] Laxmaiah A., Meshram I. I., Arlappa N. (2015). Socio-economic & demographic determinants of hypertension & knowledge, practices & risk behaviour of tribals in India. *Indian Journal of Medical Research*.

[14] Williams E. A., Keenan K. E., Ansong D. (2013). The burden and correlates of hypertension in rural Ghana: a cross-sectional study. *Diabetes & Metabolic Syndrome: Clinical Research & Reviews*.

[15] Temmar M., Labat C., Benkhedda S. (2007). Prevalence and determinants of hypertension in the Algerian Sahara. *Journal of Hypertension*.

[16] Addo J., Amoah A. G. B., Kwadwo K. A. (2006). The changing patterns of hypertension in Ghana: a study of four rural communities in the Ga district. *Ethnicity & Disease*.

[17] Maracy M. R., Feizi A., Bagherynejad M. (2012). The prevalence and correlated determinants of hypertension and type 2 diabetes: a large community-based study in Isfahan, Iran. *Pakistan Journal of Medical Sciences*.

[18] Lloyd-Sherlock P., Beard J., Minicuci N., Ebrahim S., Chatterji S. (2014). Hypertension among older adults in low and middle-income countries: prevalence, awareness and control. *International Journal of Epidemiology*.

